# Accumulation in the Organism of Bromide of Potassium

**Published:** 1889-12-07

**Authors:** 


### ACCUMULATION IN THE ORGANISM OF BROMIDE OF POTASSIUM.

Dr. Maurice Doyon reports a curious and instructive case of a girl aged twelve, who had been for some time taking three to six grains of bromide daily on account of severe epilepsy. During an attack of scarlatina she fell into a deep stupor, and attributing it to the effect of the drug he ordered its discontinuance, but was soon compelled to resume it by the advent of acute mania. A few weeks later she developed violent whooping cough, with extreme dyspnœa and cyanosis. This ended fatally, and a post-mortem examination of the organs showed the presence of bromide in all the organs, 0·7 gram being found in the liver and no less than two grams in the brain. There can be no doubt as to the somnolence during the scarlatina having been due to this, which probably also aggravated the dyspnœa in the whooping cough.

